# Supplementary material for: Pol θ-mediated end-joining uses microhomologies containing mismatches
Source: Nat Commun. 2025 Jul 2;16:6085. doi: 10.1038/s41467-025-61258-3 (PMC12222517; doi:10.1038/s41467-025-61258-3)
Supplement: Supplementary file 1 — Supplementary Information [file 41467_2025_61258_MOESM1_ESM.pdf]

Supplementary information

Pol θ-mediated end-joining uses microhomologies containing mismatches

Yuzhen Li, Ngoc K. Dang, Wei He, Mark Returan, Denisse Carvajal-Maldonado, Adele T. Guerin, Han Xu, Bin Liu, Richard D. Wood*

Department of Epigenetics and Molecular Carcinogenesis, MD Anderson Cancer Center, Houston, TX, United States.

*Corresponding author: rwood@mdanderson.org

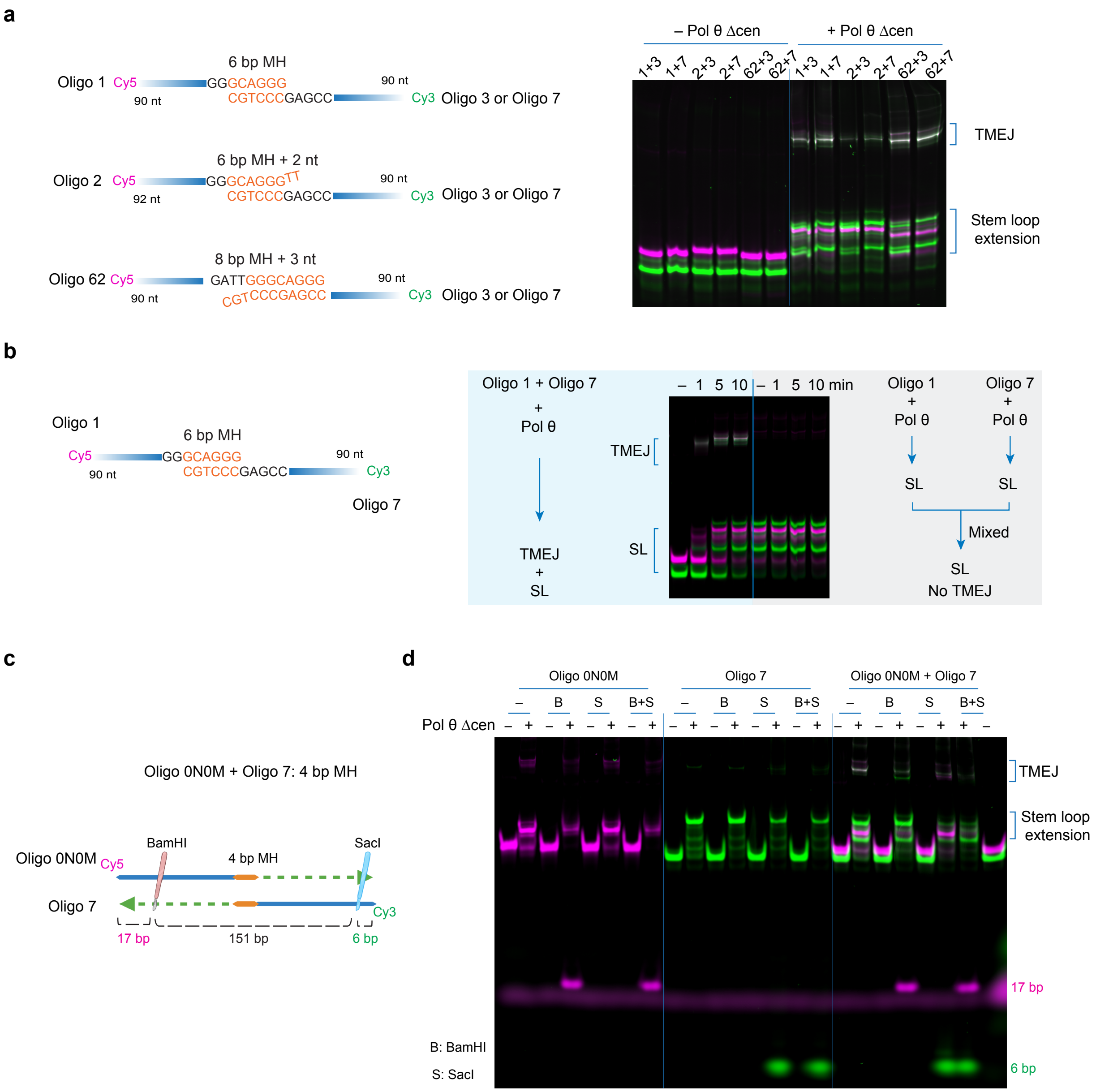
a
Oligo 1 Cy5
90 nt
6 bp MH
GGGCAGGG
CGTCCCGAGCC
90 nt
Cy3 Oligo 3 or Oligo 7
Oligo 2 Cy5
92 nt
6 bp MH + 2 nt
GGGCAGGGTT
CGTCCCGAGCC
90 nt
Cy3 Oligo 3 or Oligo 7
Oligo 62 Cy5
90 nt
8 bp MH + 3 nt
GATTGGGCAGGG
CGTCCCGAGCC
90 nt
Cy3 Oligo 3 or Oligo 7
– Pol θ Δcen
+ Pol θ Δcen
1+3 1+7 2+3 2+7 62+3 62+7 1+3 1+7 2+3 2+7 62+3 62+7
TMEJ
Stem loop extension
b
Oligo 1
6 bp MH
Cy5
90 nt
GGGCAGGG
CGTCCCGAGCC
90 nt
Cy3
Oligo 7
Oligo 1 + Oligo 7
+
Pol θ
TMEJ
+
SL
– 1 5 10 – 1 5 10 min
TMEJ
SL
Oligo 1
+
Pol θ
SL
Oligo 7
+
Pol θ
SL
Mixed
SL
No TMEJ
c
Oligo 0N0M + Oligo 7: 4 bp MH
Oligo 0N0M Cy5
BamHI
4 bp MH
SacI
Oligo 7
Cy3
17 bp
151 bp
6 bp
d
Oligo 0N0M
Oligo 7
Oligo 0N0M + Oligo 7
– B S B+S – B S B+S – B S B+S
Pol θ Δcen
TMEJ
Stem loop extension
17 bp
6 bp
B: BamHI
S: SacI

Supplementary Figure 1. Pol θ mediated end joining with ssDNAs harboring terminal or internal MHs.

a. End joining of paired or terminally unpaired oligonucleotides by Pol θ Δcen. The schematic shows different sets of oligonucleotides containing terminal or internal 6 or 8 bp MH. Electrophoresis of end joining reaction mixtures (15 min) on a native 10% polyacrylamide gel from a single experiment. Oligo 3 and Oligo 7 differ by only one base.

b. The schematic shows oligonucleotides containing terminal 6 bp MH. Electrophoresis of end joining reaction mixtures with two oligonucleotides and Pol θ Δcen from a single experiment. The blue shaded area shows reactions were initiated once Pol θ Δcen and two oligonucleotides were mixed in reaction buffer (gel lanes 1-4). The gray shaded area shows reactions where each oligonucleotide was individually incubated with Pol θ Δcen in reaction buffer for 10 min and then reactions were initiated by mixing preincubated reactions together (gel lanes 5-8).

c. Verification of end joining products with BamHI and SacI digestion. BamHI site, SacI site and fragments size of end joining products after digestion are labeled with the corresponding fluorescence color. The figure was prepared with BioRender.com and licensed for publication.

d. Electrophoresis of end joining and stem loop extension reaction mixtures with or without BamHI or SacI digestion on a native 10% polyacrylamide gel from a single experiment.

Source data are provided as a Source Data file.

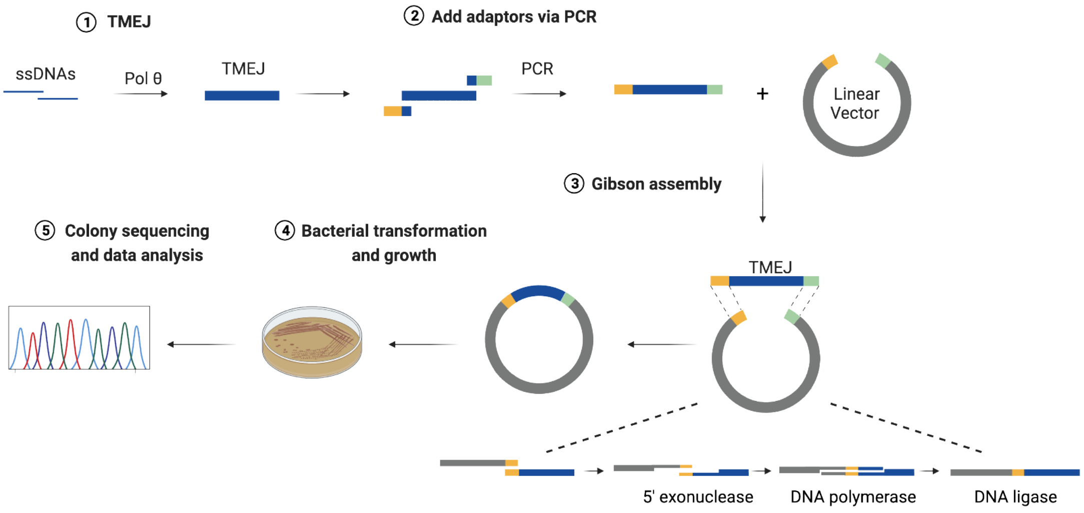

Supplementary Figure 2. Small-scale DNA sequencing scheme to characterize TMEJ products.

The strategy employs Gibson assembly followed by sequencing of assembled plasmids propagated in individual *E. coli* colonies. Created in BioRender. Li, Y. (2025) https://BioRender.com/vxre3ij. Details in Supplementary Methods.

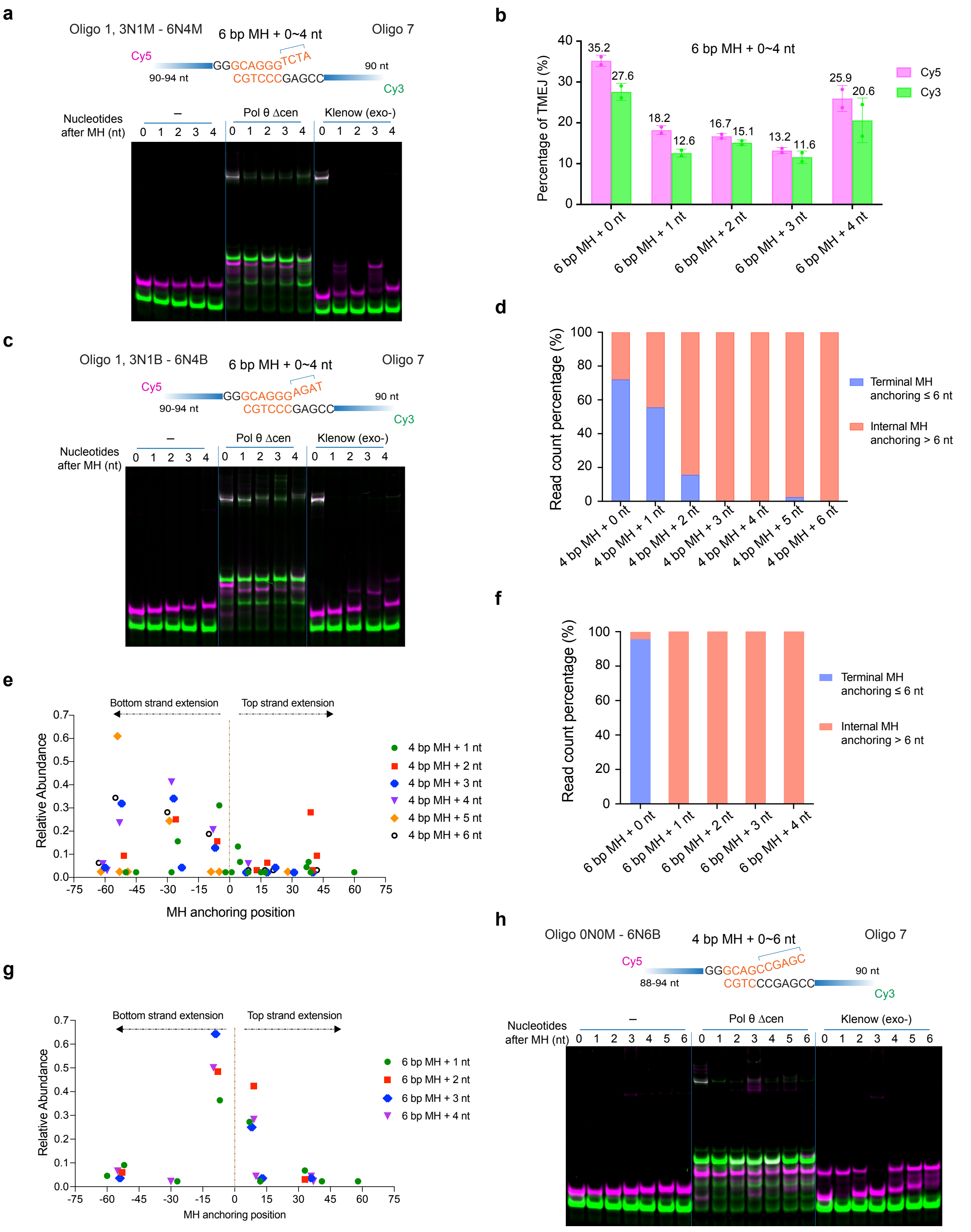
a
Oligo 1, 3N1M - 6N4M
6 bp MH + 0~4 nt
Oligo 7
Cy5
GGGCAGGGTCTA
90-94 nt
90 nt
CGTCCCGAGCC
Cy3
Nucleotides after MH (nt)
–
Pol θ Δcen
Klenow (exo-)
0 1 2 3 4
b
6 bp MH + 0~4 nt
Percentage of TMEJ (%)
35.2
27.6
18.2
12.6
16.7
15.1
13.2
11.6
25.9
20.6
Cy5
Cy3
6 bp MH + 0 nt
6 bp MH + 1 nt
6 bp MH + 2 nt
6 bp MH + 3 nt
6 bp MH + 4 nt
c
Oligo 1, 3N1B - 6N4B
6 bp MH + 0~4 nt
Oligo 7
Cy5
GGGCAGGGAGAT
90-94 nt
90 nt
CGTCCCGAGCC
Cy3
Nucleotides after MH (nt)
–
Pol θ Δcen
Klenow (exo-)
0 1 2 3 4
d
Read count percentage (%)
Terminal MH anchoring ≤ 6 nt
Internal MH anchoring > 6 nt
4 bp MH + 0 nt
4 bp MH + 1 nt
4 bp MH + 2 nt
4 bp MH + 3 nt
4 bp MH + 4 nt
4 bp MH + 5 nt
4 bp MH + 6 nt
e
Bottom strand extension
Top strand extension
Relative Abundance
MH anchoring position
4 bp MH + 1 nt
4 bp MH + 2 nt
4 bp MH + 3 nt
4 bp MH + 4 nt
4 bp MH + 5 nt
4 bp MH + 6 nt
f
Read count percentage (%)
Terminal MH anchoring ≤ 6 nt
Internal MH anchoring > 6 nt
6 bp MH + 0 nt
6 bp MH + 1 nt
6 bp MH + 2 nt
6 bp MH + 3 nt
6 bp MH + 4 nt
g
Bottom strand extension
Top strand extension
Relative Abundance
MH anchoring position
6 bp MH + 1 nt
6 bp MH + 2 nt
6 bp MH + 3 nt
6 bp MH + 4 nt
h
Oligo 0N0M - 6N6B
4 bp MH + 0~6 nt
Oligo 7
Cy5
GGGCAGCCGAGC
88-94 nt
90 nt
CGTCCCGAGCC
Cy3
Nucleotides after MH (nt)
–
Pol θ Δcen
Klenow (exo-)
0 1 2 3 4 5 6

Supplementary Figure 3. Mismatched ends promote internal, imperfectly paired MH selection by Pol θ.

a. Pol θ Δcen-mediated end joining of paired oligonucleotides with a designed core 6 bp MH followed by 0 - 4 nt mismatches. The schematic shows oligonucleotides Oligo 1 and Oligo 3N1M - 6N4M paired with Oligo 7. Electrophoresis of 10 min end joining reaction mixtures separated on a native 10% polyacrylamide gel.

b. Percentage of TMEJ products of each oligonucleotide in panel **a**. The intensity was measured with ImageJ and the graph was prepared with Prism (n = 2).

c. Pol θ Δcen mediated end joining with paired oligonucleotides with a designed core 6 bp MH followed by 0 - 4 nt mismatches. The schematic shows oligonucleotides Oligo 1 and Oligo 3N1B - 6N4B paired with Oligo 7. Electrophoresis of 10 min end joining reaction mixtures separated on a native 10% polyacrylamide gel from a single experiment.

d. Quantification of the usage of terminal and internal MHs obtained by sequencing the end-joining products shown in Fig 3**b**. A MH is designated as terminal if the anchoring position is within 6 nt at the 3' end of the ssDNA, and designated internal if the anchoring position is > 6 nt.

e. Relative abundance of MHs anchoring positions, derived from sequencing the end-joining products shown in Fig 3**b**.

f. Quantification of the usage of terminal and internal MHs obtained by sequencing the end-joining products shown in part **a**. A MH is designated as terminal if the anchoring position is within 6 nt at the 3' end of the ssDNA, and designated internal if the anchoring position is > 6 nt.

g. Relative abundance of MHs anchoring positions, derived from sequencing the end-joining products shown in panel **a**.

h. Pol θ Δcen-mediated end joining of paired oligonucleotides with a designed core 4 bp MH followed by 0 - 6 nt mismatches. The schematic shows oligonucleotides Oligo 0N0M - 6N6B paired with Oligo 7. Electrophoresis of 10 min end joining reaction mixtures separated on a native 10% polyacrylamide gel from a single experiment.

Source data are provided as a Source Data file.

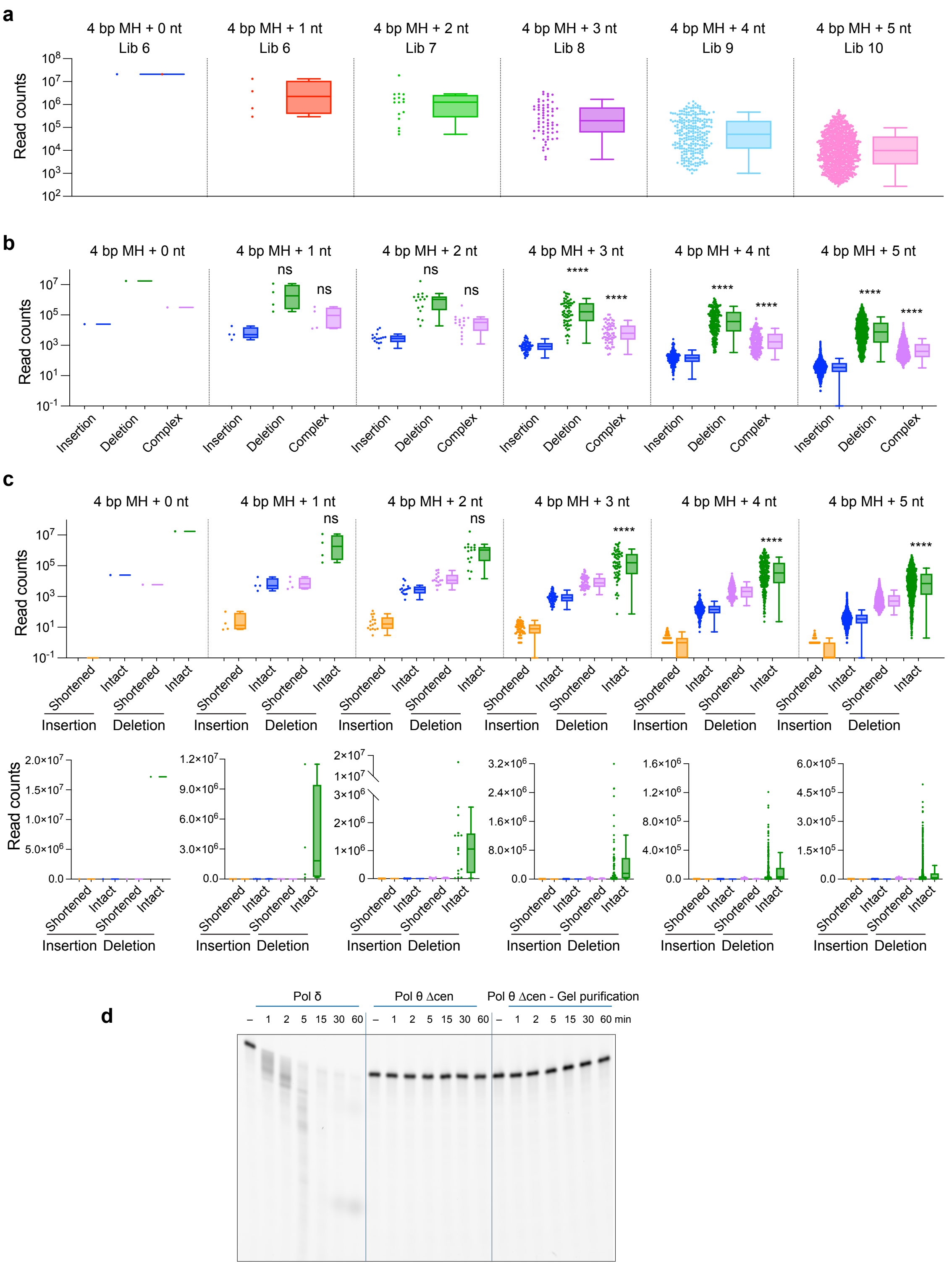
a
4 bp MH + 0 nt
Lib 6
4 bp MH + 1 nt
Lib 6
4 bp MH + 2 nt
Lib 7
4 bp MH + 3 nt
Lib 8
4 bp MH + 4 nt
Lib 9
4 bp MH + 5 nt
Lib 10
Read counts
b
4 bp MH + 0 nt
4 bp MH + 1 nt
4 bp MH + 2 nt
4 bp MH + 3 nt
4 bp MH + 4 nt
4 bp MH + 5 nt
ns
****
Read counts
Insertion
Deletion
Complex
c
4 bp MH + 0 nt
4 bp MH + 1 nt
4 bp MH + 2 nt
4 bp MH + 3 nt
4 bp MH + 4 nt
4 bp MH + 5 nt
ns
****
Read counts
Shortened
Intact
Insertion
Deletion
d
Pol δ
Pol θ Δcen
Pol θ Δcen - Gel purification
– 1 2 5 15 30 60
– 1 2 5 15 30 60
– 1 2 5 15 30 60 min

Supplementary Figure 4. Deletions are the major outcome of Pol θ mediated end joining.

a. Total end joining read counts (log scale) for the indicated oligo libraries with a blocked bottom oligonucleotide (Oligo 10). Dots indicate the individual read counts, and the box plot shows the distribution.

b. End joining read counts (log scale). Summary of insertions, deletions and complex events for the indicated oligo libraries with a blocked bottom oligonucleotide (Oligo 10). Dots indicate the individual read counts, and the box plot shows the distribution.

c. End joining read counts for insertion and deletion outcomes with shortened or intact 3' ends, for the indicated oligo libraries with a blocked bottom oligonucleotide (Oligo 10). Read count number shown with log scale (top) and linear scale (bottom). Dots indicate the individual read counts, and the box plot shows the distribution.

d. No detectable exonuclease activity in different preparations of purified Pol θ Δcen. Pol δ was used as the positive control. Oligo 6N4B was incubated with Pol θ Δcen or Pol δ for different times at 37 ^{o}C. The reaction mixtures were run on a denaturing 15% polyacrylamide gel. n = 2.

Box plots in panel a - c show the median (centre line), the 25th and 75th percentiles (bounds of box), and Tukey whiskers (1.5X interquartile range). The statistical analysis was performed using a paired two-tailed t-test. Significance is indicated as follows: ns (not significant, $p > 0.05$), * ($p \leq 0.05$), ** ($p \leq 0.01$), *** ($p \leq 0.001$) and **** ($p \leq 0.0001$).

Source data are provided as a Source Data file.

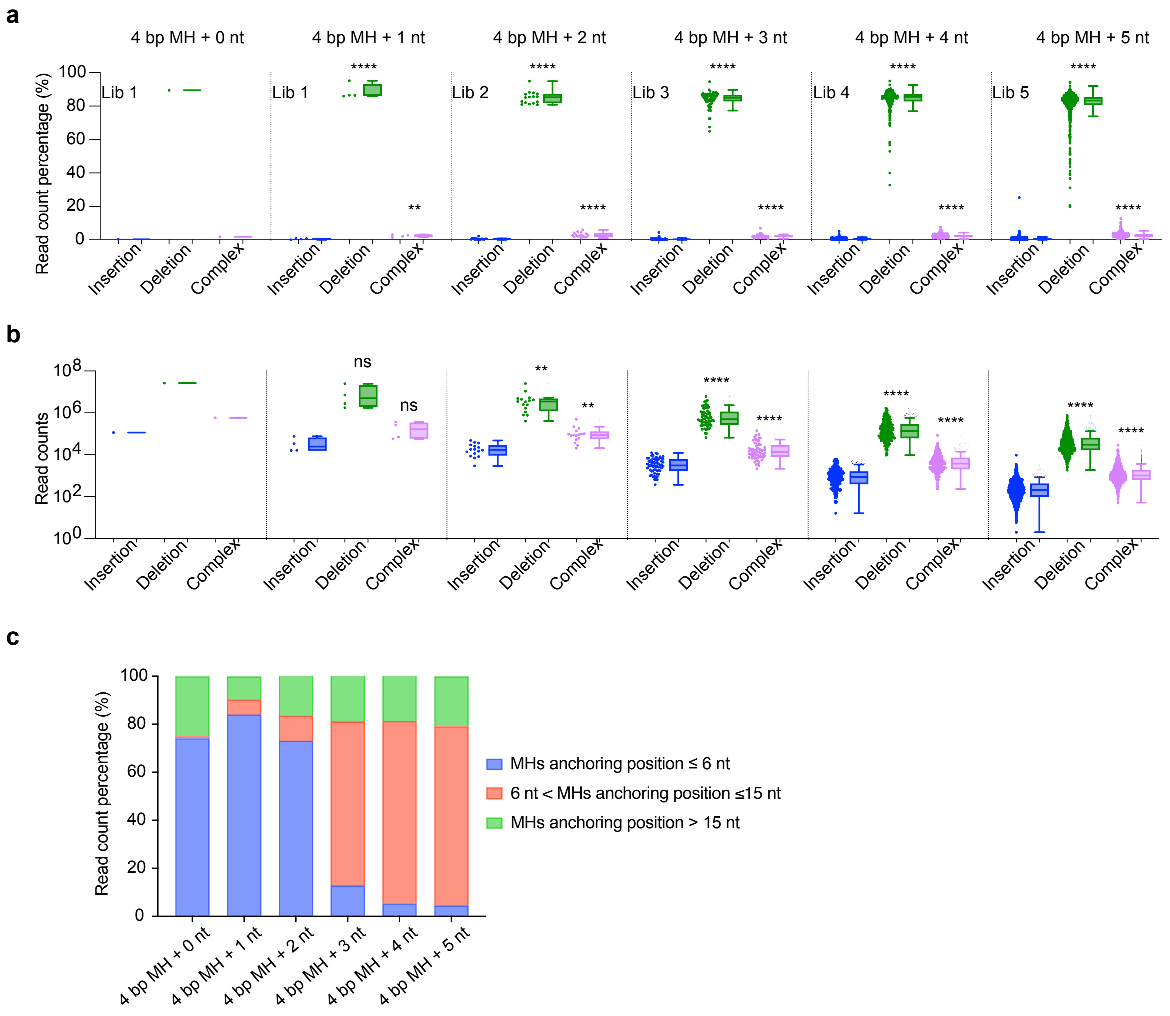

Supplementary Figure 5. Deletions are the major outcome (unblocked bottom strand).

a. End joining read count percentage for insertions, deletions, and complex events for the indicated top oligo libraries with an unblocked bottom oligonucleotide (Oligo 8), shown as percentage. Dots indicate the individual read counts percentage, and the box plot shows the distribution.

b. End joining read counts as in *a*, plotted with log scale. Dots indicate the individual read counts, and the box plot shows the distribution.

c. End joining read count percentage of MHs anchored at different regions for the indicated libraries with an unblocked bottom oligonucleotide (Oligo 8).

Box plots in panel a - b show the median (centre line), the 25th and 75th percentiles (bounds of box), and Tukey whiskers (1.5X interquartile range). Statistical analysis was performed using a paired t-test. Significance is indicated as follows: ns (not significant, $p > 0.05$), * ($p \leq 0.05$), ** ($p \leq 0.01$), *** ($p \leq 0.001$) and **** ($p \leq 0.0001$). Source data are provided as a Source Data file.

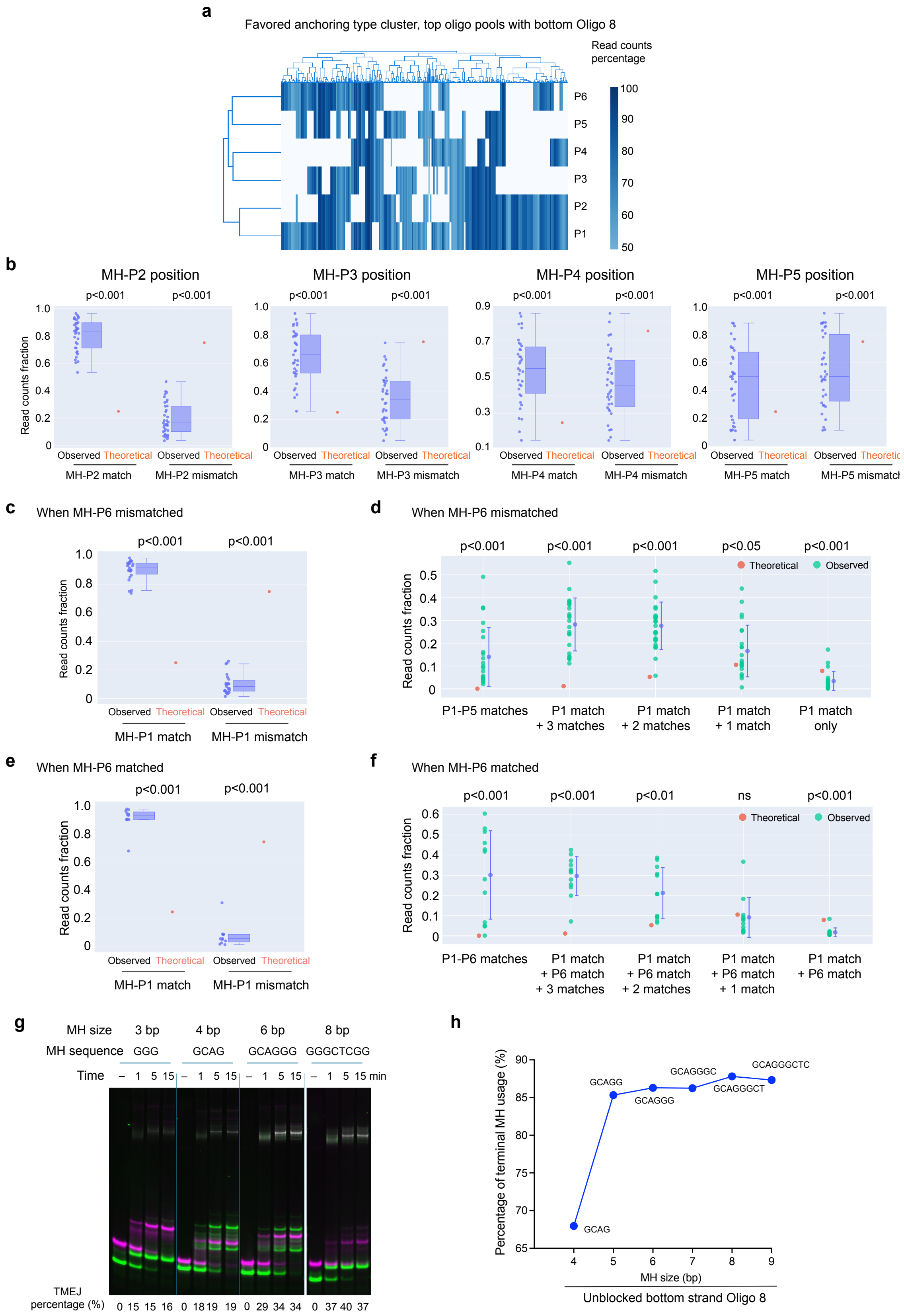
a
Favored anchoring type cluster, top oligo pools with bottom Oligo 8
Read counts percentage
P6
P5
P4
P3
P2
P1
b
MH-P2 position
MH-P3 position
MH-P4 position
MH-P5 position
p<0.001
Read counts fraction
Observed Theoretical
MH-P2 match
MH-P2 mismatch
MH-P3 match
MH-P3 mismatch
MH-P4 match
MH-P4 mismatch
MH-P5 match
MH-P5 mismatch
c
When MH-P6 mismatched
MH-P1 match
MH-P1 mismatch
d
When MH-P6 mismatched
p<0.05
Theoretical
Observed
P1-P5 matches
P1 match + 3 matches
P1 match + 2 matches
P1 match + 1 match
P1 match only
e
When MH-P6 matched
f
When MH-P6 matched
p<0.01
ns
P1-P6 matches
P1 match + P6 match + 3 matches
P1 match + P6 match + 2 matches
P1 match + P6 match + 1 match
P1 match + P6 match
g
MH size 3 bp 4 bp 6 bp 8 bp
MH sequence GGG GCAG GCAGGG GGGCTCGG
Time – 1 5 15 – 1 5 15 – 1 5 15 – 1 5 15 min
TMEJ percentage (%) 0 15 15 16 0 18 19 19 0 29 34 34 0 37 40 37
h
Percentage of terminal MH usage (%)
GCAG
GCAGG
GCAGGG
GCAGGGC
GCAGGGCT
GCAGGGCTC
MH size (bp)
Unblocked bottom strand Oligo 8

Supplementary Figure 6. A terminal match and additional matches are important for selection of MH by Pol θ.

a. Plot of the favored MH used by Pol θ Δcen for end joining reactions. All anchoring positions for Library 1-5 oligonucleotide pools (unblocked bottom "Oligo 8") were analyzed. If an oligonucleotide used the same MH in more than 50% of the read counts, it was designated a favored MH. Each column corresponds to one of these 832 individual oligonucleotides having a favored MH. Rows correspond to position P1 to P6 of the MH as in Fig 6**a.** The percentage of matching at each position in the favored MH is indicated with a *blue* color scale. Mismatched positions are *white*.

b. Matching at position P2, P3, P4 or P5 of a MH is favored by Pol θ for anchoring and extension during initiation of end joining in Library 10. The fraction of matches or mismatches at each position (*blue dots and box plot*) is compared with theoretical fractions (*red dots*). The statistical significance is labeled with the p value derived from a two-sided t-test.

c. Matching at position P1 of a MH is favored by Pol θ for anchoring and extension during initiation of end joining when position P6 is mismatched in Library 10. The observed P1 match or mismatch fraction is compared with theoretical fractions with two-sided t-test and the statistical significance was labeled with p value.

d. MH with more matches show higher anchoring frequencies than theoretical frequencies when position P6 is mismatched in Library 10. The comparison was done with two-sided t-test and the statistical significance was labeled with p value.

e. Matching at position P1 of a MH is favored by Pol θ for anchoring and extension during initiation of end joining when position P6 is matched in Library 10. The observed P1 match or mismatch fraction is compared with theoretical fractions with two-sided t-test and the statistical significance was labeled with p value.

f. MH with two or more additional matches show higher anchoring frequencies than theoretical frequencies when position P6 is matched in Library 10. The comparison was done with two-sided t-test and the statistical significance was labeled with p value.

g. Pol θ Δcen mediated end joining of different paired oligonucleotides with designed terminal 3, 4, 6 and 8 bp MH. The terminal MH sequences are labeled at the top of the figure. Percentage of TMEJ products was labeled under each corresponding lane. n = 2. The intensity was measured with ImageJ.

h. The anchoring data for specific oligonucleotides with the indicated terminal MH sequences (4 - 9 bp), complementary to bottom oligonucleotide Oligo 8, were extracted from the high-throughput sequencing data set. The percentage of terminal MH usage is shown for each sequence.

Box plots in panel b, c and e show the median (centre line), the 25th and 75th percentiles (bounds of box), and Tukey whiskers (1.5X interquartile range). Source data are provided as a Source Data file.

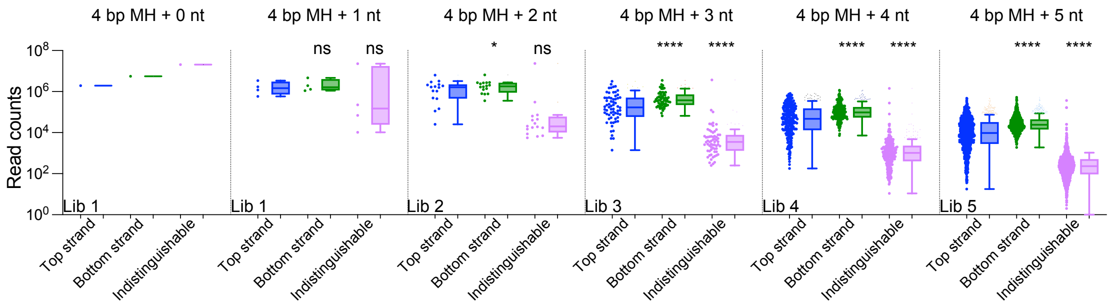

Supplementary Figure 7. Pol θ usually extends one single stranded oligonucleotide during end joining.

End joining read counts (log scale) relevant to Fig 7**a** (where read counts are plotted as %). Dots indicate the individual read counts, and the box plot shows the distribution. Box plots show the median (centre line), the 25th and 75th percentiles (bounds of box), and Tukey whiskers (1.5X interquartile range). The statistical analysis was performed using a paired t-test. Significance is indicated as follows: ns (not significant, $p > 0.05$), * ($p \leq 0.05$), ** ($p \leq 0.01$), *** ($p \leq 0.001$) and **** ($p \leq 0.0001$). Source data are provided as a Source Data file.

a

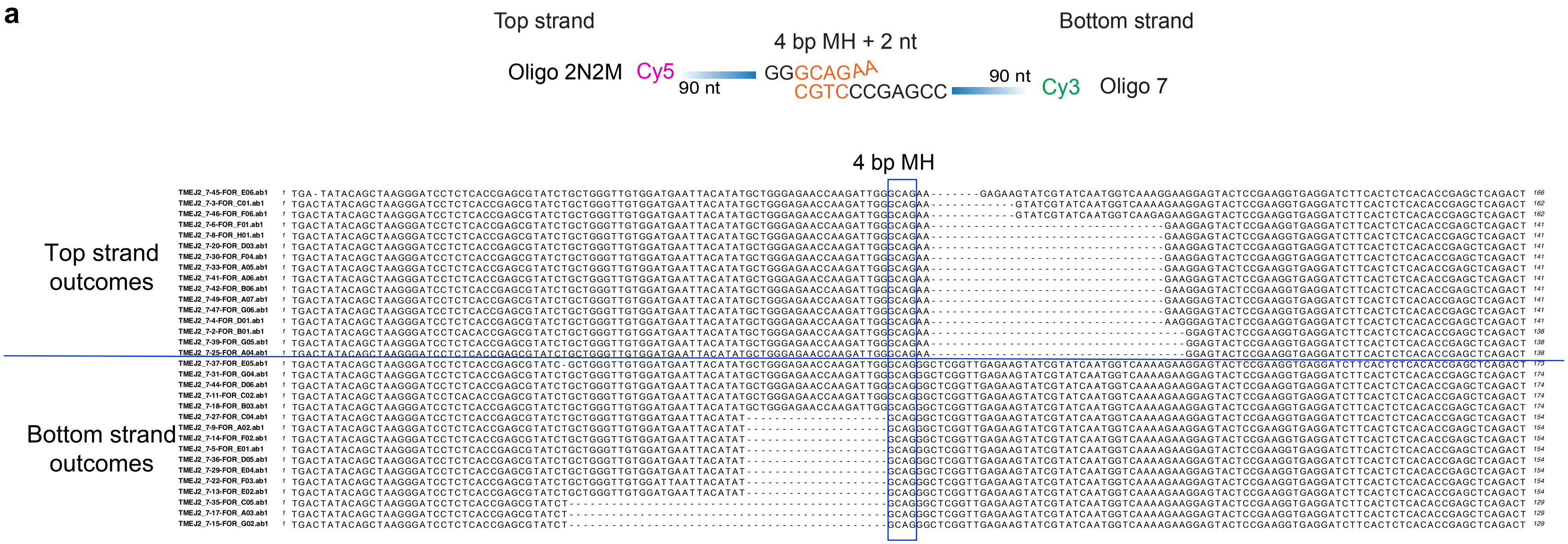
Top strand
Bottom strand
4 bp MH + 2 nt
Oligo 2N2M
Cy5
90 nt
GGGCAGAA
CGTCCCGAGCC
90 nt
Cy3
Oligo 7
4 bp MH
Top strand outcomes
Bottom strand outcomes

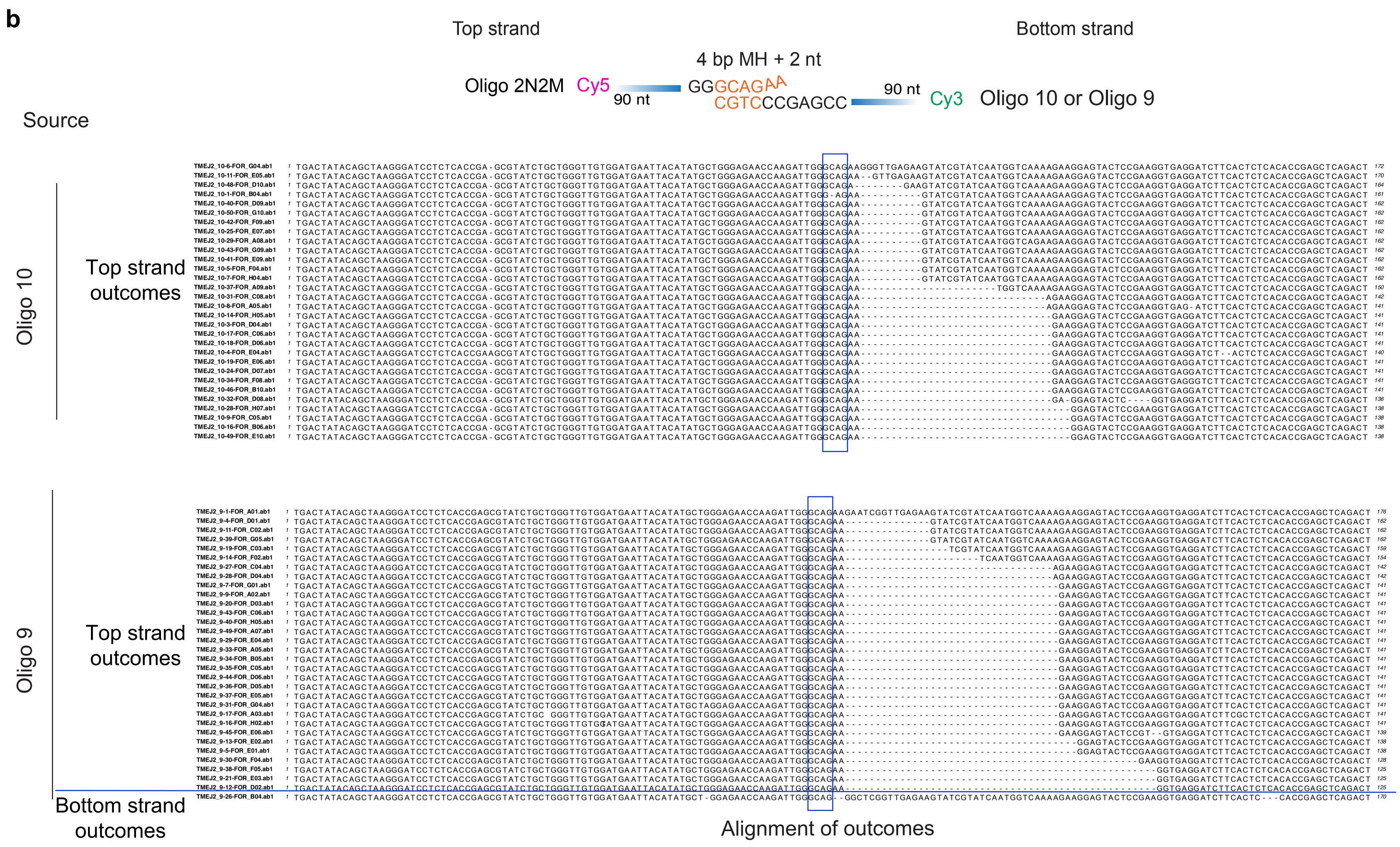

b
Top strand
Bottom strand
4 bp MH + 2 nt
Oligo 2N2M
Cy5
90 nt
GGGCAGAA
CGTCCCGAGCC
90 nt
Cy3
Oligo 10 or Oligo 9
Source
Oligo 10
Top strand outcomes
Oligo 9
Top strand outcomes
Bottom strand outcomes
Alignment of outcomes

c

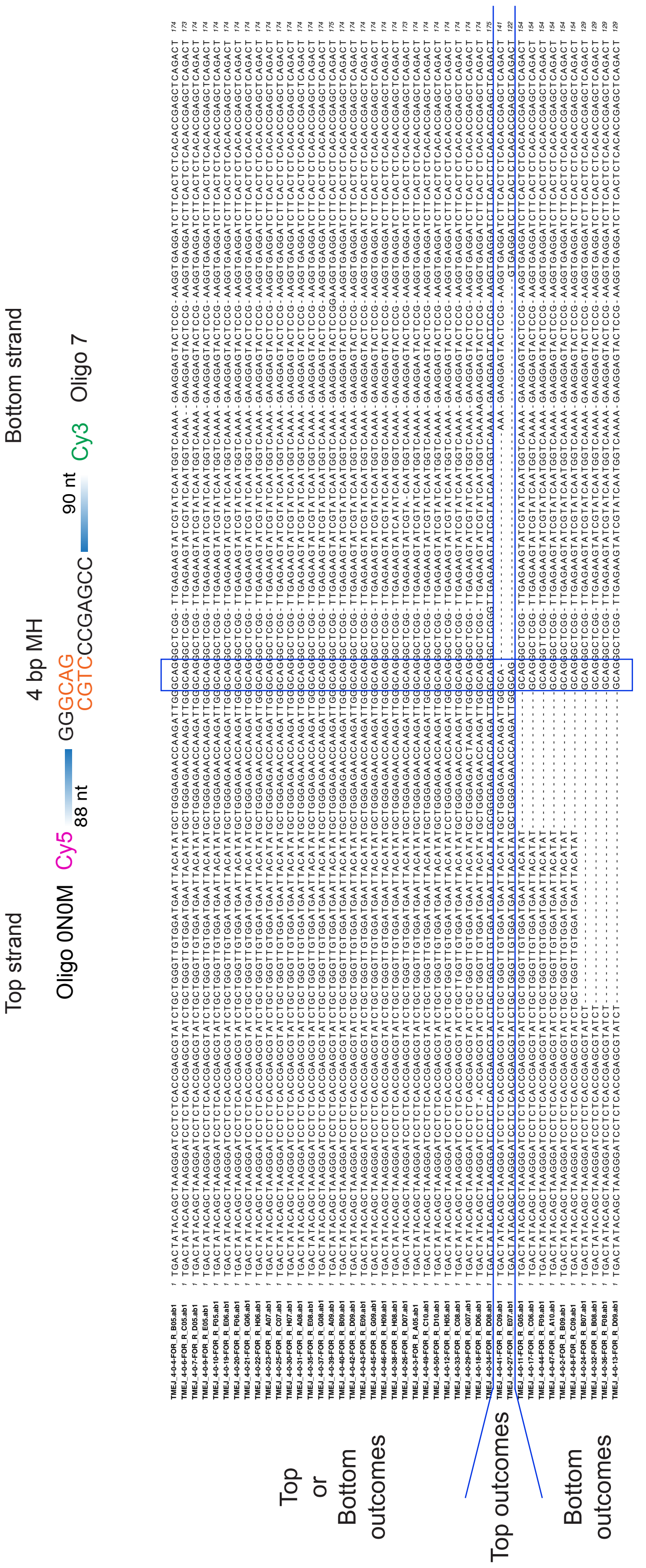
Top strand
Bottom strand
Oligo 0N0M
Cy5
88 nt
GGGCAG
CGTCCCGAGCC
4 bp MH
90 nt
Cy3
Oligo 7
Top or Bottom outcomes
Top outcomes
Bottom outcomes

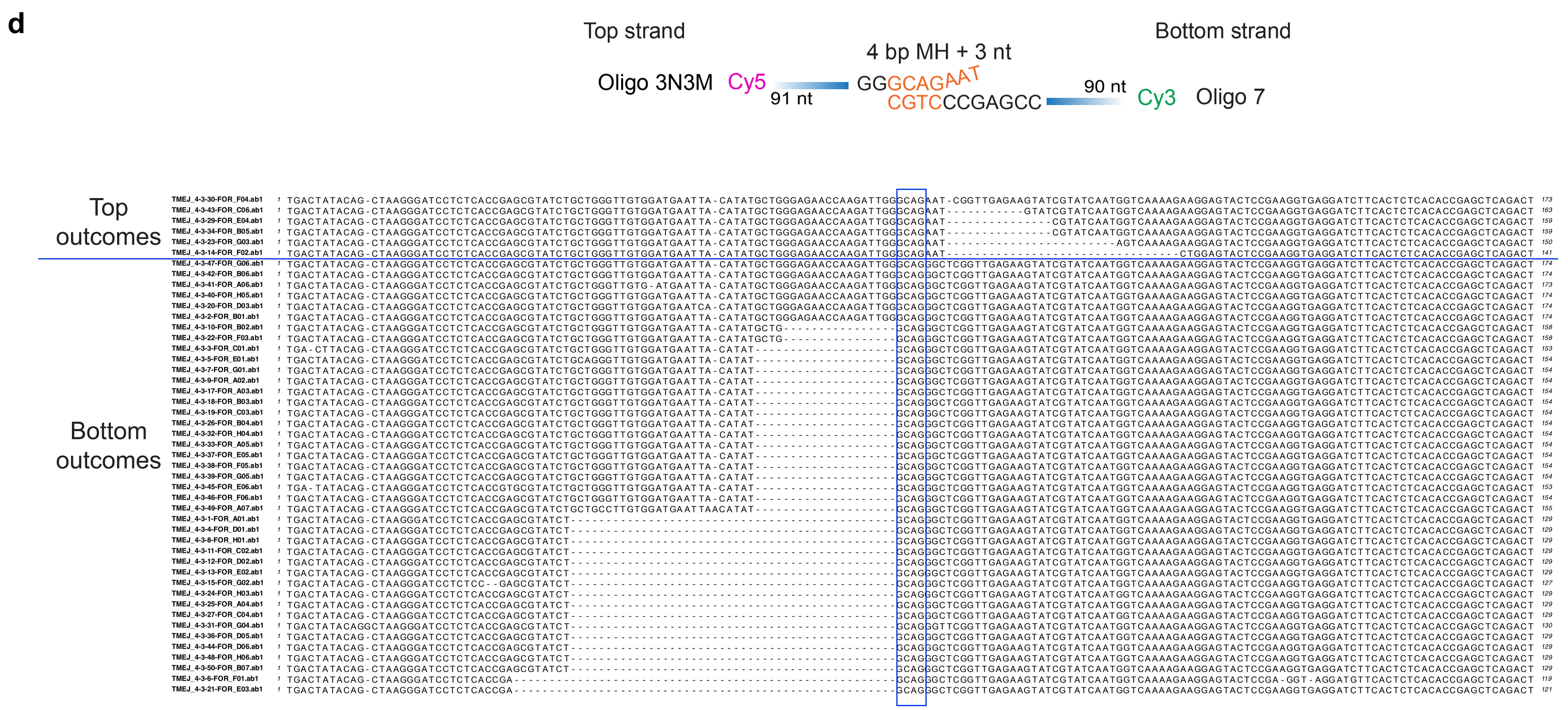
d
Top strand
Bottom strand
4 bp MH + 3 nt
Oligo 3N3M
Cy5
91 nt
GGGCAGAAT
CGTCCCGAGCC
90 nt
Cy3
Oligo 7
Top outcomes
Bottom outcomes

Supplementary Figure 8. Larger typeface version of the alignments.

a. Alignment in Fig 2**a**.

b. Alignment in Fig 2**d**.

c. Alignment in Fig 3**c**.

d. Alignment in Fig 3**d**.

Source data are provided as a Source Data file.

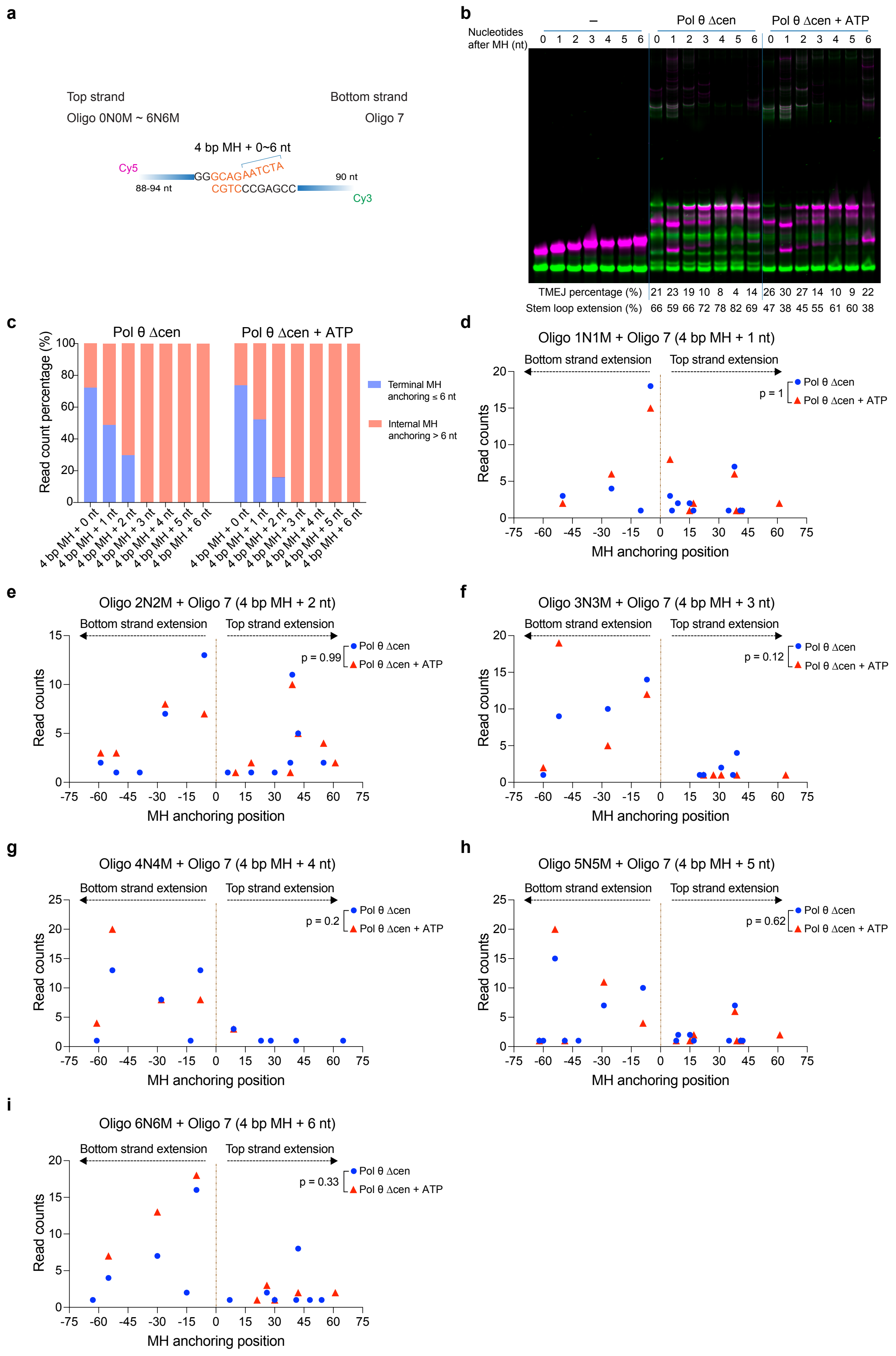
a
Top strand
Oligo 0N0M ~ 6N6M
Bottom strand
Oligo 7
4 bp MH + 0~6 nt
Cy5
88-94 nt
GGGCAGAATCTA
CGTCCCGAGCC
90 nt
Cy3
b
Nucleotides after MH (nt)
–
Pol θ Δcen
Pol θ Δcen + ATP
0 1 2 3 4 5 6 0 1 2 3 4 5 6 0 1 2 3 4 5 6
TMEJ percentage (%) 21 23 19 10 8 4 14 26 30 27 14 10 9 22
Stem loop extension (%) 66 59 66 72 78 82 69 47 38 45 55 61 60 38
c
Pol θ Δcen
Pol θ Δcen + ATP
Read count percentage (%)
Terminal MH anchoring ≤ 6 nt
Internal MH anchoring > 6 nt
4 bp MH + 0 nt
4 bp MH + 1 nt
4 bp MH + 2 nt
4 bp MH + 3 nt
4 bp MH + 4 nt
4 bp MH + 5 nt
4 bp MH + 6 nt
d
Oligo 1N1M + Oligo 7 (4 bp MH + 1 nt)
Bottom strand extension
Top strand extension
p = 1
Pol θ Δcen
Pol θ Δcen + ATP
Read counts
MH anchoring position
e
Oligo 2N2M + Oligo 7 (4 bp MH + 2 nt)
p = 0.99
f
Oligo 3N3M + Oligo 7 (4 bp MH + 3 nt)
p = 0.12
g
Oligo 4N4M + Oligo 7 (4 bp MH + 4 nt)
p = 0.2
h
Oligo 5N5M + Oligo 7 (4 bp MH + 5 nt)
p = 0.62
i
Oligo 6N6M + Oligo 7 (4 bp MH + 6 nt)
p = 0.33

Supplementary Figure 9. ATP has no effect on MH selection.

a. Schematic of oligonucleotides Oligo 0N0M – 6N6M paired with Oligo 7.

b. Pol θ Δcen-mediated end joining of paired oligonucleotides with a core 4 bp MH followed by 0 – 6 nt, performed in the presence or absence of ATP. Electrophoresis of 15 min end joining reaction mixtures was separated on a native 10% polyacrylamide gel. n = 2. The percentage of TMEJ and stem loop extension products is labeled under corresponding lanes.

c. Quantification of the usage of terminal and internal MHs obtained by sequencing the end-joining products shown in panel **b**. A MH is designated as terminal if the anchoring position is within 6 nt at the 3' end of the ssDNA, and designated internal if the anchoring position is > 6 nt.

d-i. Read counts of different MHs anchored by Pol θ of paired oligonucleotides with a core 4 bp MH followed by 0 – 6 nt, derived from sequencing the end-joining products shown in panel **b**. The distributions are statistically no different as indicated by the labeled p value derived from Kolmogorov-Smirnov test (KS-test).

Source data are provided as a Source Data file.

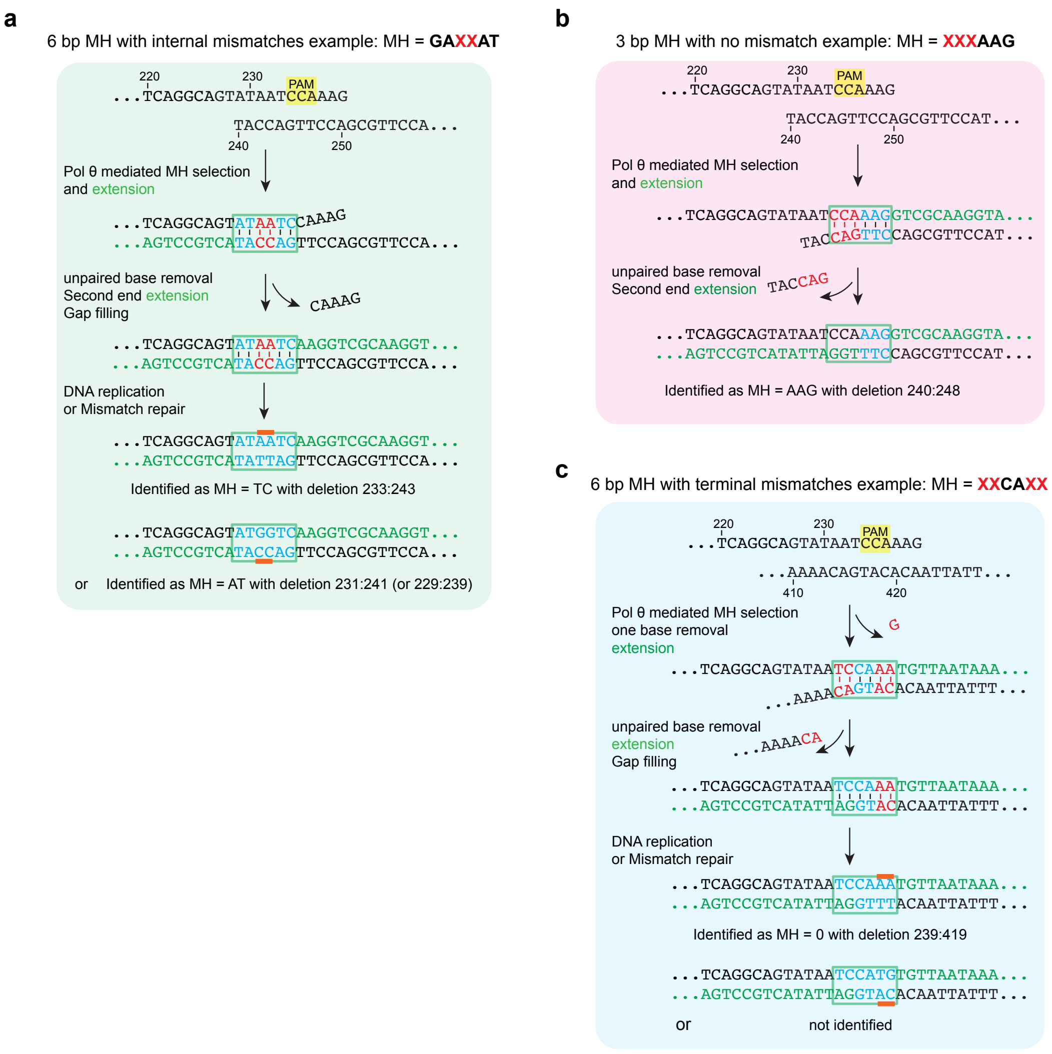

Supplementary Figure 10. MH containing mismatches are selected during TMEJ *in vivo*.

The local sequence environments are shown for DSB repair outcomes following introduction of a DSB in the human *HPRT* gene by CRISPR-Cas9, as tabulated in Supplementary Table 3 of Hwang *et al.*[1].

a. An 11 bp deletion (233-243) occurring only in *POLQ*-proficient cells. The MH is scored as 2 bp MH by traditional analysis, and as 6 bp MH (green box) with two mismatches (red nucleotides) by the revised criteria suggested here. The bottom strand may extend as shown. The top strand is processed by nuclease activity before or after bottom strand extension. Following mismatch repair, two outcomes would arise, both of them represented in the data set.

b. A 9 bp deletion (240-248) occurring only in *POLQ*-proficient cells. This occurs as a 3 bp MH by traditional analysis, as well as by the revised criteria suggested here (green box). The top strand may extend as shown with the bottom strand processed by nuclease activity before or after bottom strand extension.

c. A 181 bp deletion (230-419) occurring only in *POLQ*-proficient cells. This was scored as 0 MH but may be reconsidered as containing two base pairs at positions P3 and P4. If the bottom strand is processed by nuclease activity, it could then extend and give outcomes that would be scored as a 0 bp MH (or not identified) after mismatch repair.

Supplementary Methods

Data analysis.

The NGS sequencing data in this manuscript were analyzed with the High Performance Computing facility at MD Anderson (HPC) and have been deposited in the NCBI Sequence Read Archive (SRA) (accession number PRJNA1178638). Libraries 1 - 5 were prepared with the top oligo pools (Oligo 0N-5N) and normal (unblocked) bottom strand (Oligo 8), and Libraries 6 - 10 use the same top oligo pools with a blocked bottom strand (Oligo 10). The reads are demultiplexed into different libraries with the designed indexes and the quality of raw sequences was checked by FastQC (http://www.bioinformatics.babraham.ac.uk/projects/fastqc/). The raw paired reads were merged with PEAR [2]. The merged sequence reads were further demultiplexed with barcodes corresponding to the 0N - 5N random nucleotides at 3′-terminals of the top oligos. A total of 2730 demultiplexed files were generated. To avoid demultiplexing errors, two additional flanking nucleotides on each side of the barcodes were included in the searching query.

- **Libraries 6 - 10:**

Libraries 6 - 10 were analyzed by the pipeline described in Supplementary Fig 11. Reads with sequence length less than the minimum length of the designed oligos (88 nucleotides) are filtered as short errors. Using merged top and bottom oligos as the reference sequence, pairwise global alignments with the scoring settings (match:1, mismatch: 0, gap open penalty: -4 and gap extension penalty: -2) identifies three additional types of errors:

1. Unclassified error: Reads have more than 100 identical perfect alignments with the reference sequences.
2. Primer error: Reads contain indels within the PCR primer region.
3. Strand error: Reads contain an indel or more than one mutation within the first 84 bases (the synthesized oligo sequences before the core 4 bp MH).

The three types of errors may be caused by alignment limitation, sequencing errors or mutations generated by PCR. The stats for error reads in Libraries 6 - 10 are summarized in Supplementary Fig 12.

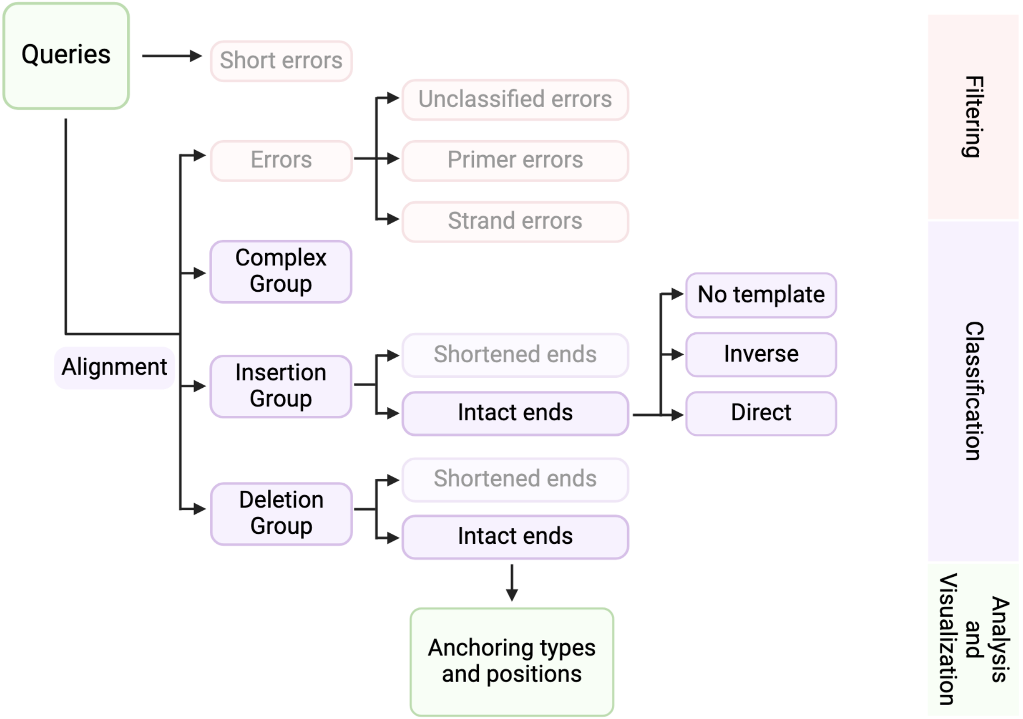

Supplementary Figure 11. Data analysis pipeline for samples in Libraries 6 - 10.

After filtering for errors, queries are classified into read groups (purple boxes), and then the deletion group (comprising the majority of reads) is used to determine anchoring types and positions.

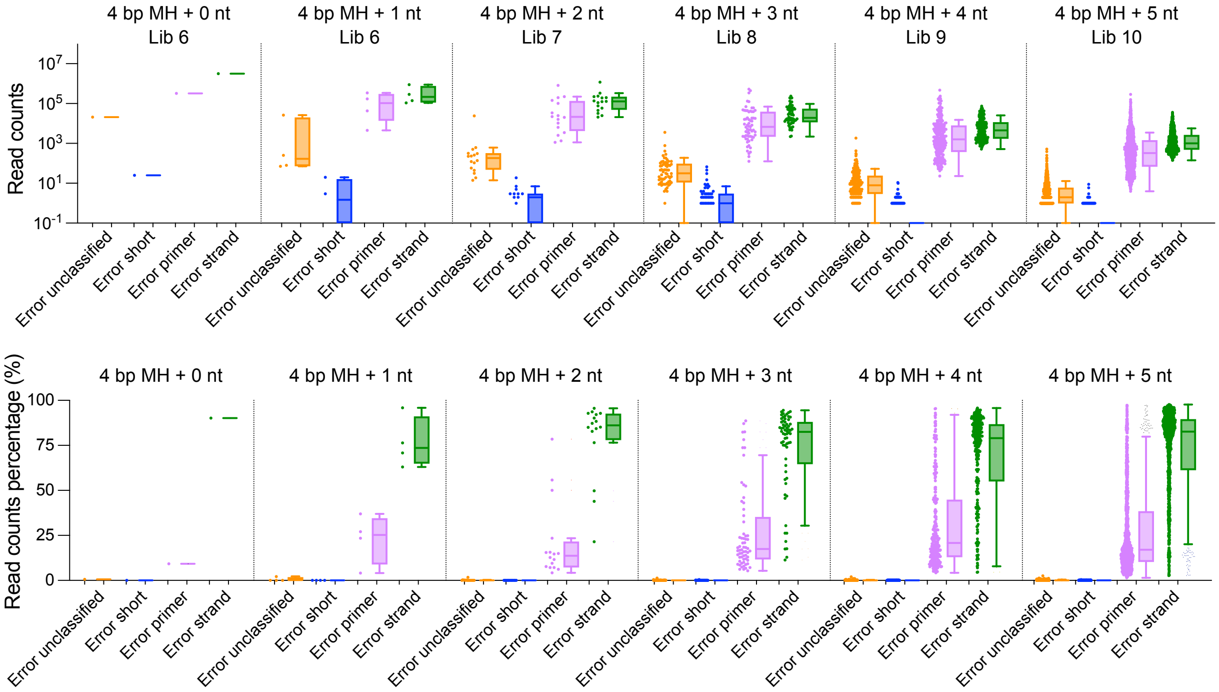

Supplementary Figure 12. Error read counts and percentage of Libraries 6 - 10. Each top oligo library (4 bp MH + 0 - 5 nt) contains 1, 4, 16, 64, 256 and 1024 individual oligonucleotide outcomes, respectively. Box plots show the median (centre line), the 25th and 75th percentiles (bounds of box), and Tukey whiskers (1.5X interquartile range). Source data are provided as a Source Data file.

After filtering, the remaining reads were further grouped according to the status of insertion and deletion identified. The status is characterized by the join region which starts from the 3′ end of the top oligo and extends to the position where five consecutive paired bases first appear after alignment with the reference sequence. Only query sequences including intact 3′ ends of the reference were kept, as Pol θ does not possess exonuclease activity (Supplementary Fig 4c -d).

1. Complex Group: The join region contains a gap ≥ 2 bases, or mismatch ≥ 2 bases.
2. Insertion Group: The read alignment finds a gap on the reference. The Insertion Group was further broken down into three subgroups: reverse repeat (reverse complement of an insertion matching the reference), direct repeat (an insertion mapped to the extended region of the oligos) and no template insertions (Insertions not in previous two subgroups).
3. Deletion Group: The read alignment finds a gap on the query sequences. Reads from the Deletion Group were subjected to downstream anchoring analysis. The deletion size equals the MH anchoring distance defined in Fig 5**b**. We defined encoding characters (Supplementary Data 1) to denote different types of pairing. This allows a matrix to be generated representing the anchoring types and positions for each extended oligonucleotide.

- ### Libraries 1 - 5

Since the 3′ end of the bottom oligo in Libraries 1 - 5 is not blocked by 3′-phosphate as it is in Libraries 6-10, either top or bottom oligos may be extended by Pol θ Δcen. A modified analytical

pipeline is described in Supplementary Fig 13. Pairwise global alignment has assorted the query reads into four classes: Top Strand Read Class, Bottom Strand Read Class, Indistinguishable Read Class and Unknown-error Read Class.

1. Top Strand Read Class: Query reads are from the top oligo extension with the intact 3′-end (from the 79th nucleotide to 3′-end of the top oligo).
2. Bottom Strand Read Class: Query reads are bottom oligo extension with the intact 3′-end (the last 10 nucleotides matching 3′-end of the bottom oligo complementary sequence).
3. Indistinguishable Read Class: Sequences of the query reads can't provide information if a top oligo or a bottom oligo is used for the extension.
4. Unknown-error Read Class: Query reads contains neither of two intact 3′ tails.
   Similar to Libraries 6 - 10, Top Strand Read Class and Bottom Strand Read Class can be further assorted into three different groups: Complex Group, Insertion Group and Deletion Group after primer errors and strand errors are filtered. The Deletion Group is subject to downstream anchoring analysis similar to Libraries 6 - 10.

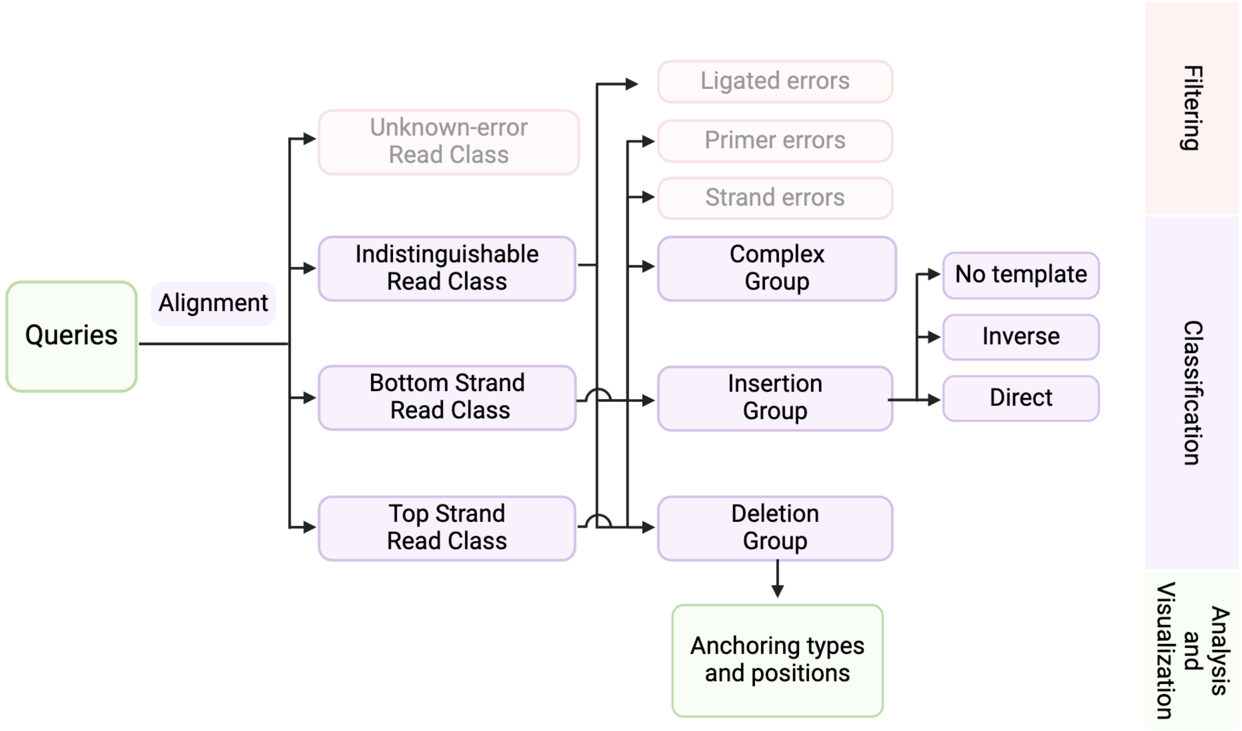

Supplementary Figure 13. Data analysis pipeline for samples in Libraries 1 - 5.

After filtering for errors, queries are classified into read groups (purple boxes), and then the deletion group (comprising the majority of reads) is used to determine anchoring types and positions.

○ Cosine correlation

The Cosine correlation of the MH anchoring matrix for each top strand outcome with unique random MH was calculated between Libraries 1 - 5 and Libraries 6 - 10 (Fig 4**c**). Some outcomes in the Deletion Group are not included in the correlation analysis because they are indistinguishable due to end-joining from terminal MHs.

○ Clustering analysis

For Fig 6**c** and supplementary Fig 6**a**, clustering plots of were generated by unsupervised agglomerative clustering with the sklearn cluster algorithm.

Supplementary Table 1. Primers for PCR

Name	Sequence 5' → 3'	Length (nt)
R1/2	AAACGACGGCCAGTGAGTCTGAGCTCGGTGTGAG	34
F1	GCCTGCAGGTCGACTTGACTATACAGCTAATGGATCCTC	39
F2	GCCTGCAGGTCGACTTGACTATACAGCTAAGGGATCC	37
F-Seq	CACTTTATGCTTCCGGCTCG	20
ASP-ForL	ACACTCTTTCCCTACACGACGCTCTTCCGATCTTGACTATACAGCTAAGGGATCC	55
ASP-RewL	GTGACTGGAGTTCAGACGTGTGCTCTTCCGATCTTCCTCACCTTCGGAGTACTCCTT	57

Supplementary References

1. Hwang, T. et al. Defining the mutation signatures of DNA polymerase theta in cancer genomes. *NAR Cancer* **2**, zcaa017 (2020).
2. Zhang, J., Kobert, K., Flouri, T. & Stamatakis, A. PEAR: a fast and accurate Illumina Paired-End reAd mergeR. *Bioinformatics* **30**, 614-20 (2014).
