# Supplementary material for: Pol θ-mediated end-joining uses microhomologies containing mismatches
Source: Nat Commun. 2025 Jul 2;16:6085. doi: 10.1038/s41467-025-61258-3 (PMC12222517; doi:10.1038/s41467-025-61258-3)
Supplement: Supplementary file 2 — Description of Additional Supplementary Files [file 41467_2025_61258_MOESM2_ESM.pdf]

Description of Additional Supplementary Files

File name: Supplementary Data 1

Description: High throughput sequencing related material information including each oligo sequence with barcode for oligo pools, bottom strand sequence, index for each library and 24 characters encoding.

File name: Supplementary Data 2

Description: Oligonucleotides for small-scale TMEJ experiments.
